# Morphology study of zinc anode prepared by electroplating method for rechargeable Zn-MnO_2_ battery

**DOI:** 10.1016/j.heliyon.2019.e02681

**Published:** 2019-10-21

**Authors:** Nattaporn Chaba, Sutasinee Neramittagapong, Arthit Neramittagapong, Nawapak Eua-anant

**Affiliations:** a Department of Chemical Engineering, Faculty of Engineering, Khon Kaen University, Khon Kaen, 40002, Thailand; b Research Center for Environmental and Hazardous Substance Management (EHSM), Khon Kaen University, Khon Kaen, 40002, Thailand; c Department of Computer Engineering, Faculty of Engineering, Khon Kaen University, Khon Kaen, 40002, Thailand

**Keywords:** Chemistry, Chemical engineering, Materials science, Chemical energy storage, Materials characterization, Electrochemical engineering, Energy storage technology, Mossy structures, Anode electrode, Zinc anode, Dendrite

## Abstract

Zinc electrodes prepared by electrodepositing zinc on a copper plate in ZnSO_4_ electrolyte were studied to determine the most suitable condition of zinc anode preparation for high performance Zn-MnO_2_ battery. Deposition of zinc on substrate material was confirmed using X-ray diffraction (XRD) measurement. Morphological characterization of zinc anode was performed by scanning electron microscopy (SEM). It was observed that concentration of electrolyte and electrical current density influenced the morphology of zinc electrode. At 1 M ZnSO_4_ and current density values of 0.06–0.1 A/cm^2^, it was found that the morphological structure of zinc electrode was orderly arranged in a layer-by-layer structure. This indicated that the current density played an important role on deposition morphology and even on the crystal structure. Performance of electrodes was tested by a battery analyzer for 100 cycles. The results of the tests showed that the electrode with the layer-by-layer morphology yielded a high efficiency of up to 99.97% which was higher and more stable than those of the electrodes with disordered and scattered morphology. The layer-by-layer morphology is therefore a key factor for improving the performance of Zn-MnO_2_ cell.

## 1 Introduction

Rechargeable batteries are necessary for storing electricity generated from solar power systems. The battery used for solar energy storage is a lead-acid battery which is expensive and not durable causing a very high cost for solar energy application. Lead-acid batteries contain lead – a heavy metal – which is toxic and hazardous to the environment. Currently, lead has been replaced by zinc in electrode production, as it is more affordable, safe, and environmentally friendly [1, 2, 3]. However, there are limitations in utilizing zinc as an electrode. After being used for a long time, zinc electrodes will give rise to sprouting dendrites, which cause short circuits and shorten battery life [4]. Typical methods used for fabricating zinc electrodes are electroplating on a metal substrate and forming the electrodes from zinc powder [5, 6]. It has been reported that electroplating on metal substrate is an easy way to prepare electrodes and is not as complicated as forming the electrodes from zinc powder. There are crucial factors which must be considered in electrodeposition on a metal substrate since they affect the performance of batteries. A study by Alias et al. [7], using ZnSO_4_ at 1 M concentration and a current density between 0.01-0.1 A/cm^2^, found that the current used in electroplating affected the performance of electroplating itself. In addition, there is a study [8] in changing the shape of zinc electrode by performing an electroplating process on flat sheets and using a ratio of utilized current to limited current, and the result of the study showed that the zinc electrode had grown like moss when the current ratio was below 0.4. Furthermore, it was also found that zinc deposits on the electrodes were arranged as solid crystals when the current ratio was increased from 0.4 to 0.9. More studies [9, 10] on the morphology of zinc electrode found that if the morphology of zinc was arranged evenly and packed, it would result in a better performance of battery cell. Therefore, in this research we aimed to determine the most suitable condition for electrodeposition on the copper substrate in order to prepare an electrode for battery by utilizing ZnSO_4_ as an electrolyte. Effects of current density and concentration of electrolyte, as well as the time used in electrodeposition on the morphology of zinc and the performance of battery equipped with prepared electrodes was investigated.

## 2 Materials and methods

### 2.1 Zinc anode preparation

Zinc anode was prepared by an electroplating method. Copper sheet of 0.3 mm thickness (Sincharoen Metal, Bangkok, Thailand) was cut to small pieces with a size of 1 × 2.5 cm^2^. It was polished by using fine sandpaper (No.360, TOA) and was soaked in acetone for 10 minutes and then started the electroplating process by a power supply (GPC-3030D). 99.9998% pure Zinc for electroplating (Samliam Chromium, Thailand) and using 200 cm^3^ ZnSO_4_ (RCI Labscan) as an electrolyte, the reaction occurs as follows:

(1) Cathode: Zn(s) → Zn^2+^(aq)+2e^−^

(2) Anode: Cu^2+^(aq)+2e^−^ → Cu(s)

As for electrodeposition on a copper substrate, the weight of copper substrate before and after plating process is needed to determine the weight of zinc deposits on a copper substrate in order to calculate the current efficiency from Faraday's Law:

(3) $$Current\;Efficiency=\;\frac{m\;}{c\;\times I\;\times t}\;\times 100\%$$

where *m* is the weight of the zinc deposits on the copper substrate (g), c is an electrochemical equivalent constant of zinc, 1.1295 g/A⋅hr, I is a current density used in plating (A), t is the plating time (hr). The electrodeposited copper substrate was analyzed for its crystal structure with scanning electron microscopy technique (SEM, S-3000N, Hitachi, Japan) and X-ray diffraction (XRD, PANalytical, EMPYREAN) to confirm that zinc was deposited on the substrate material.

MnO_2_ cathodes were prepared by hot pressing process. Nickel foam purchased from MTI Corporation was used as a substrate for cathodes. It was cut into small pieces with a dimension of 1 × 2.5 cm^2^, rinsed by deionized water, and then dried in an oven at 100 °C for 6 hrs. A mixture of 60 wt% MnO_2_ (SCI Labscan), 20 wt% graphite (SCI Labscan), 20 wt% Teflon powder (PTFE, Polytetrafluoroethylene, SCI Labscan) were pressed onto the dried nickel foam substrate at a temperature of 240 °C and a pressing pressure of 8.27 MPa. The effective mass loading of MnO_2_ after pressing was about 0.12 g/cm^2^.

### 2.2 Experiment

Our preliminary study (unpublished) showed that factors of zinc electrode preparation influencing the electrodeposition on the copper substrate were the current used in plating, the concentration of electrolyte, as well as the time taken for electrodeposition. In this experiment, 0.02–0.1 A/cm^2^ current densities, ZnSO_4_ solution at 0.1–1 M concentrations and 1 hour of plating duration were used. After that, the best experimental condition would be selected to carry out an electrodeposition process at 1, 3, 5 and 7 hours. Cycle voltammetry was performed by a battery analyzer (NEWARE Technology Co., LTD China) with the potential range of 0.6–1.8 V ​for 100 cycles per cell at the current charge-discharge rate of 50 mA and 10 ml of electrolyte. 6 M KOH with 40%ZnO was the electrolyte of the battery cell.

## 3 Results and discussion

**Fig. 1:**
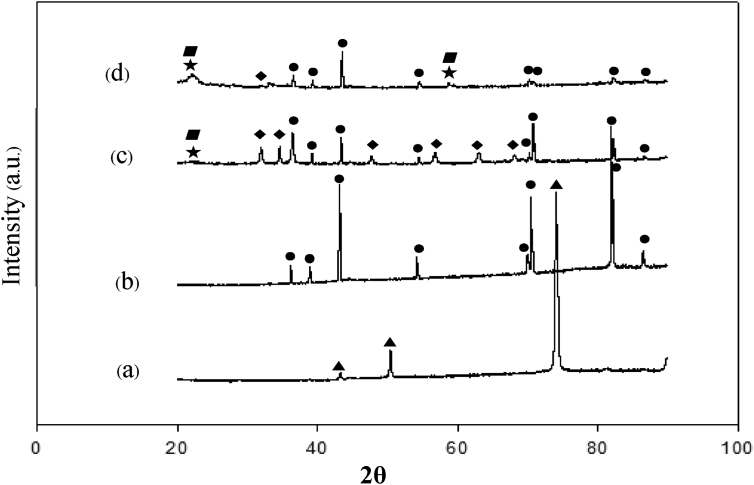
XRD patterns of (a) Cu plate, (b) Zn deposited at a current density of 0.1 A/cm^2^ with 1M ZnSO_4_ (fresh electrode), (c) Zn deposited at a current density of 0.1 A/cm^2^ with 1M ZnSO_4_ after the performance test and (d) Zn deposited at a current density of 0.1 A/cm^2^ with 0.5M ZnSO_4_ after the performance test.

Fig. 1 shows an XRD analysis of Cu substrate (Fig. 1a), Zn deposited (fresh electrode) at a current density of 0.1 A/cm^2^ with 1M ZnSO_4_ (Fig. 1b), Zn deposited (after performance test) at a current density of 0.1 A/cm^2^ with 1M ZnSO_4_ (Fig. 1c), and Zn deposited (after performance test) at a current density of 0.1 A/cm^2^ with 0.5M ZnSO_4_ (Fig. 1d). The Cu plate peaks were detected at 2θ = 44.41°, 51.72° and 76.32°, and these peaks confirmed that the substrate was copper. Fig. 1b also showed the presence of Zn which indicated that Zn was actually deposited on the copper substrate. After 100 cycles of performance test, there appeared Mn^2+^ ions which formed Zn _1-x_MnO_2_•nH_2_O and ZnMn_2_O_4_ over the Zn anode. The presence of Mn^2+^ as in this study was also reported in Ito et al [11].

**Fig. 2:**
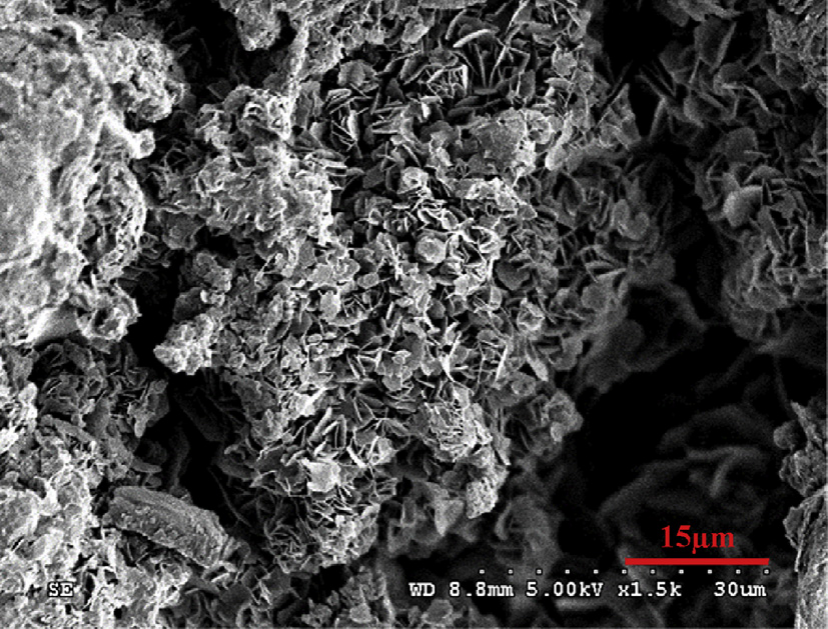
SEM image of Zn deposits at current density 0.1 A/cm^2^ with 0.1 M ZnSO_4_ for 1 hour.

**Fig. 3:**
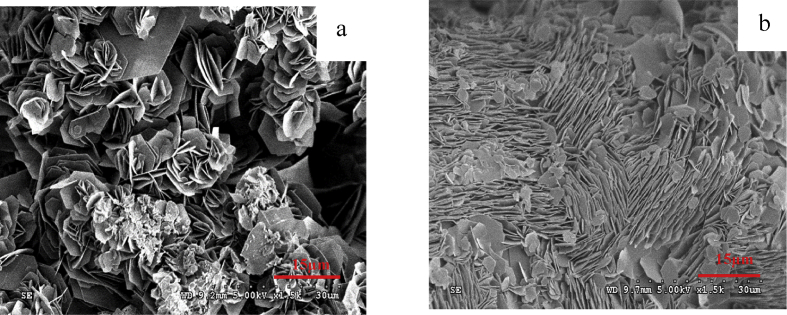
SEM image of Zn deposits with 0.5 M ZnSO_4_ for 1 hour. (a) Zn deposits at the current of 0.6 A/cm^2^ (b) Zn deposits at the current of 0.1 A/cm^2^.

**Fig. 4:**
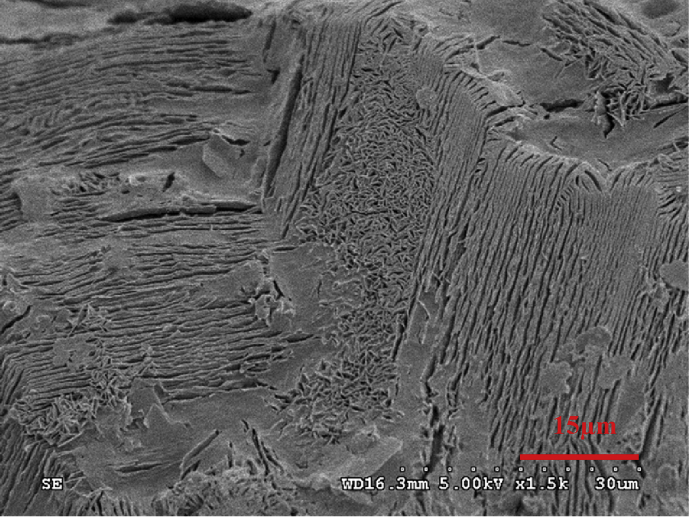
SEM image of Zn deposits at the current of 0.08–0.1 A/cm^2^ with 1 M ZnSO_4_ for 1 hour.

From the different appearance of the Zn anodes prepared using different conditions, it was thought that the morphology of the Zn deposited might also be different. The Zn anodes were therefore analyzed under SEM. An analysis of morphology of the prepared zinc anode on the copper substrate from electroplating process using ZnSO_4_ at 0.1M concentration with a current density of 0.02–0.1 A/cm^2^ for 1 hour indicated that the morphological characteristics of zinc deposits on the copper substrate were binding, and clustering in a disorderly arrangement and easy to decay as shown in Fig. 2. While increasing the concentration of ZnSO_4_ to 0.5 M with a current density at 0.02–0.06 A/cm^2^ revealed that zinc was deposited on the copper substrate in a disorderly manner as shown in Fig. 3a. However, when the current density was increased to higher values at 0.08–0.1 A/cm^2^, the morphological characteristics of zinc deposits on the copper substrate changed to a loose layer-by-layer arrangement as shown in Fig. 3b. As the concentration of ZnSO_4_ was increased to 1 M while the current density was varied between 0.08-0.1 A/cm^2^, the morphology of zinc deposits on the copper substrate were arranged as layers in a more orderly and closely packed manner as shown in Fig. 4. The current density and the concentration of electrolyte, therefore, seem to be the most prominent parameters which determine the plating current efficiency, the deposit morphology and even the crystal structure [12, 13, 14].

In addition, from our experiment it was also found that electrodeposition on a copper substrate using a low-concentration electrolyte with equal current would require a higher voltage. Even though this condition provided a higher current efficiency, the higher voltage resulted in disorderly arrangement of the zinc deposits. However, electrodeposition on a copper substrate using a high-concentration electrolyte with equal current required the voltage lower than that of the process with a low-concentration electrolyte, and resulted in a neat morphology of zinc deposits.

At a low electrolyte concentration, the morphology of zinc electrodeposits took hexagonal-like flake structure with the size of 3–10 μm.as evidenced by Fig. 2. As the electrolyte concentration was increased, the zinc deposits nucleated and grew to form polygonal Zn Plate, as depicted in Fig. 3a, and the deposits transformed to a multilayer structure when the concentration was further increased as illustrated by Fig. 4. It can be seen that zinc morphology gradually changes from dispersive nanoparticles to multilayer microparticles with hexagonal shapes when the concentration increased. The morphology change during the electrodeposition process was mainly attributed to the concentration and electrodeposition rate. The high concentration has high electrical conductivity and low resistance due to the fact that the mole conductivity of a solution depends on the ions in a solution or concentration of electrolyte.

**Fig. 5:**
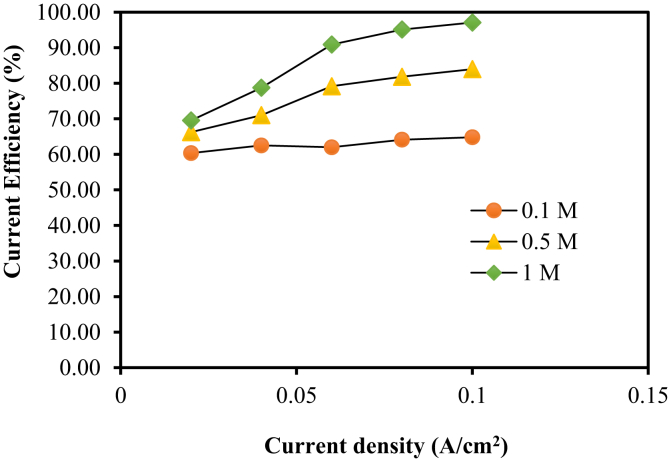
Current density and current efficiency with ZnSO_4_ solutions for 1 hour.

From the electrodeposition experiment, it was found that the amount of zinc deposit on the copper substrate varied according to the current used in plating. The current efficiency calculated using plating current density values at 0.02, 0.04, 0.06, 0.08, and 0.1 A/cm^2^, ZnSO_4_ concentration of 0.1 M for 1 hour were approximately 60.35, 62.52, 61.99, 64.12, and 64.81%, respectively, and the electrodeposition rates of each current density were 0.18, 0.38, 0.56, 0.78 and 0.98 g/hr, respectively. These electrodeposition rates correspond to the effective mass loadings of zinc deposit of 0.05, 0.12, 0.17, 0.24 and 0.30 g/hr⋅cm^2^ respectively. Meanwhile, increasing the concentration of ZnSO_4_ from 0.1M to 0.5 M while keeping the same current density values as those of the case of 0.1 M concentration resulted in 66.26, 71.01, 79.16, 81.84, and 83.97% current efficiency, and the electrodeposition rates of each current density were 0.20, 0.43, 0.72, 0.99 and 1.28 g/hr. respectively. Similarly, these electrodeposition rates correspond to the effective mass loadings of zinc deposit of 0.06, 0.13, 0.22, 0.30 and 0.40 g/hr⋅cm^2^ respectively. However, when the concentration of ZnSO_4_ was increased to 1 M with the same plating duration and the same current density values, the current efficiency values became higher at 69.54, 78.74, 90.90, 95.12, and 97.09%, respectively, as shown in Fig. 5. The electrodeposition rates of each current density were 0.21, 0.48, 0.83, 1.16 and 1.48 g/hr, respectively and the effective mass loadings of Zn deposit corresponding to these electrodeposition rates are respectively 0.06, 0.15, 0.17, 0.25 and 0.45 g/hr⋅cm^2^ as tabulated in Table 1. It is worth noting that for the different electrolyte concentrations at the current density ranging from 0.02 to 0.04 A/cm^2^, the current efficiency values were slightly different and the difference in the current efficiency values was more pronounced from the current density of 0.06 A/cm^2^ onwards. This can be explained by the fact that the rate of electron transfer, hence the rate of Zn deposit, is proportional to the current density. Furthermore, Baik and Fray [15] also claimed that high current density made the substrate surface more suitable for Zn deposit.

**Table 1:** Electroplating test data of Zn on copper substrate.

ZnSO_4_ (M)	Current Density (A/cm^2^)	Effective mass loading (g/hr⋅cm^2^)	Electrodeposition rate (g/hr.)	Efficiency Current (%)	Morphology of Zn deposits
0.1	0.02	0.05	0.18	60.35	Disorder
	0.04	0.12	0.38	62.52	Disorder
	0.06	0.17	0.56	61.99	Disorder
	0.08	0.24	0.78	64.12	Disorder
	0.10	0.30	0.98	64.81	Disorder
0.5	0.02	0.06	0.20	66.26	Disorder
	0.04	0.13	0.43	71.01	Disorder
	0.06	0.22	0.72	79.16	Disorder
	0.08	0.30	0.99	81.84	Disorder
	0.10	0.40	1.28	83.97	loose layer-by-layer
1.0	0.02	0.06	0.21	69.54	Disorder
	0.04	0.15	0.48	78.74	Disorder
	0.06	0.17	0.83	90.90	loose layer-by-layer
	0.08	0.25	1.16	95.12	loose layer-by-layer
	0.10	0.45	1.48	97.09	Layer-by-layer

**Fig. 6:**
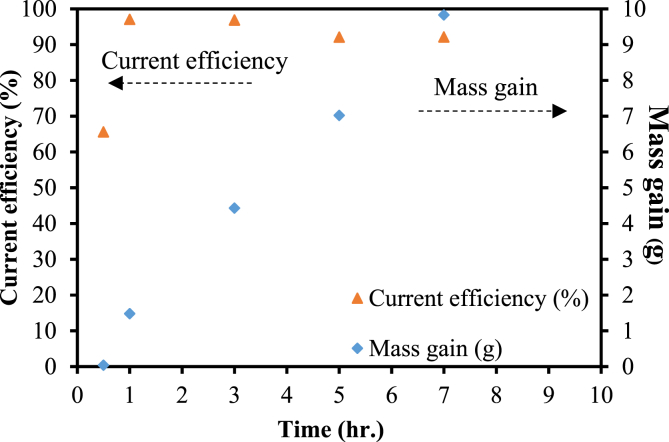
Current efficiency and mass gain of Zn deposits on copper substrate after 0.5, 1, 3, 5 and 7 hours of plating.

**Fig. 7:**
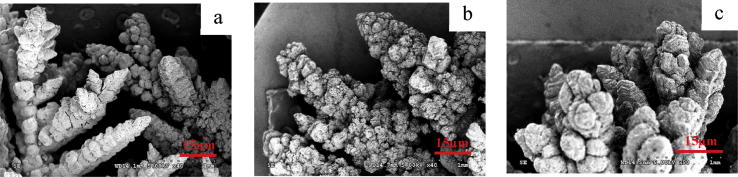
SEM image of dendrite sprouting of zinc a) plating for 3 hours, b) plating for 5 hours, c) plating for 7 hours.

When preparing zinc anode on a copper substrate using the current density of 0.1 A/cm^2^, ZnSO_4_ of 1 M, and the durations of 0.5, 1, 3, 5 and 7 hours, it was discovered that the amount of zinc deposits on the copper substrate were proportional to the time used in plating as shown in Fig. 6. It was also found that if plating duration was increased, the current efficiency would be reduced due to Zinc branches sprouting on the copper substrate as shown in Fig. 7a, b and c, respectively. Zinc began to sprout new branches approximately in the third hour of plating. Zinc sprouts are undesirable because they may cause short circuits during actual use of the battery.

**Fig. 8:**
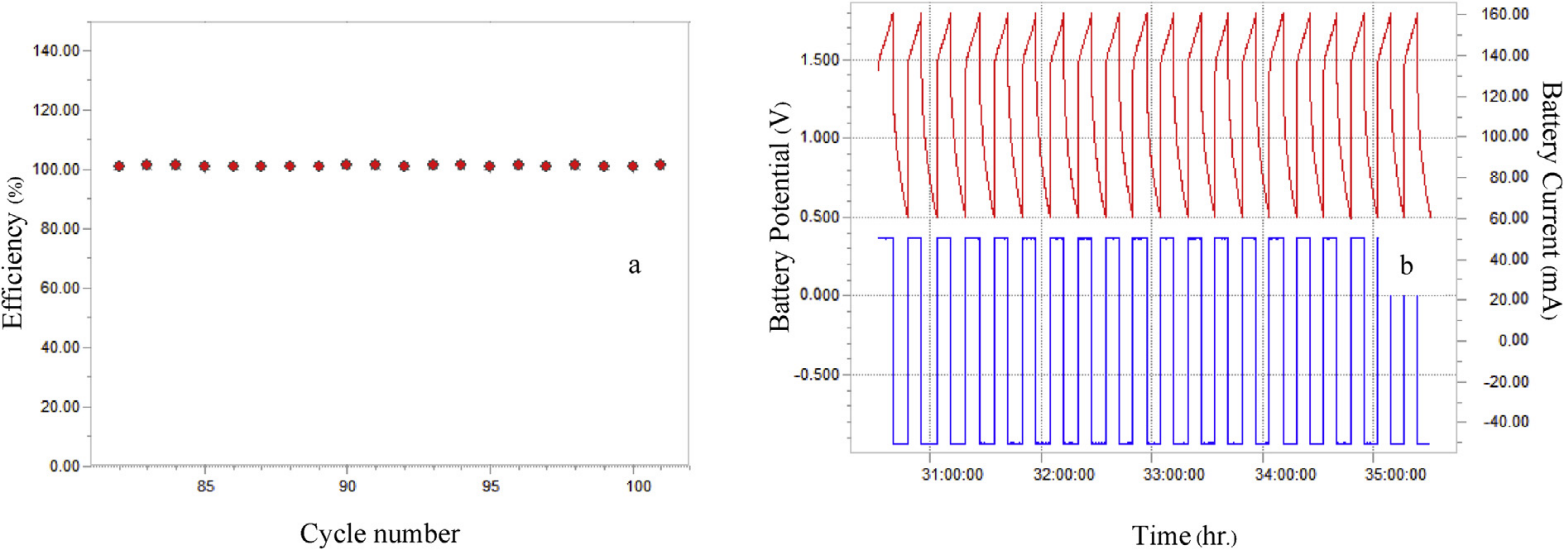
Data indicating the performance of Zn-MnO_2_ battery using Zn deposited at a current density of 0.1 A/cm^2^ with 1M ZnSO_4_ (a) efficiency of cell and (b) battery potential and battery current with time.

**Fig. 9:**
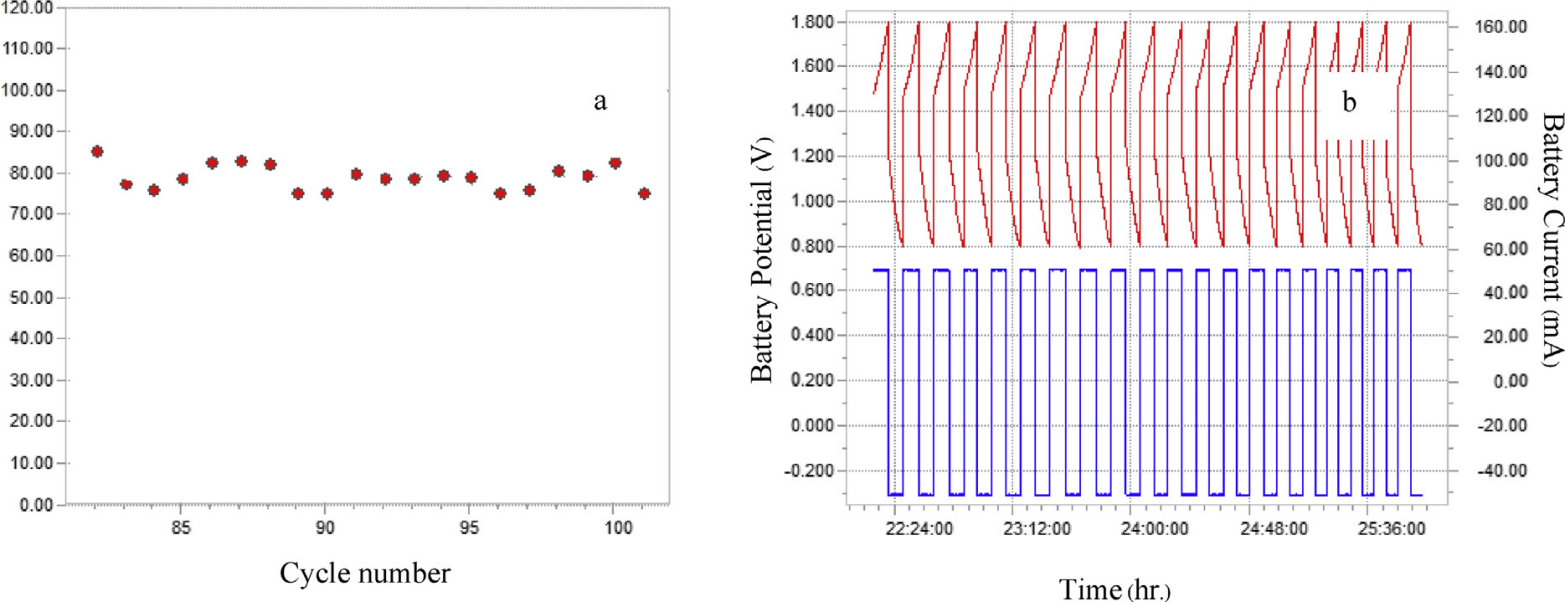
Data indicating the performance of Zn-MnO_2_ battery which Zn deposited at a current density of 0.1 A/cm^2^ with 0.5M ZnSO_4_ (a) efficiency of cell and (b) battery potential and battery current with time.

It has been reported by Desai et al. [9] and Ito et al. [10] that Zn deposit arrangement on the substrate has an important influence on the performance of the battery. In this investigation, we selected the anodes prepared under two different conditions which gave disorderly Zn deposit (0.1 A/cm^2^, 0.5 M ZnSO_4_) and which resulted in orderly Zn deposit (0.1 A/cm^2^, 1 M ZnSO_4_) for efficiency tests of Zn-MnO_2_ battery. Results of the tests of the battery cell using the prepared electrodes are shown in Figs. 8a, b and 9a, b. Zn deposited at a current density of 0.1 A/cm^2^ with 1M ZnSO_4_ was tested for 100 cycles which gave higher and more stable efficiency than those of Zn deposited at the same current density with 0.5 M ZnSO_4_. From the results, it can be inferred that the electrodes with orderly arrangement of zinc deposits are more efficient and more stable than those with disorderly zinc deposits. Therefore, disorderly morphology is thought to be a major factor which causes zinc to sprout branches easily on copper. Branching of zinc on the copper substrate can cause the battery short-circuit which shortens the battery life [4, 5, 6, 7, 8, 9].

Table 2 shows some important test results of Zn-MnO_2_ batteries by current researchers. Yinxiang et al. [16] and Donghong et al. [17] studied Zn-MnO_2_ battery with gel electrolyte. Both studies prepared the Zn anodes on nanoplates by electroplating. The test results of these studies revealed that the battery performed with high efficiency and stability, comparable with our results even though their Zn morphological structure was arranged in a freestanding manner. Although the Zn deposit on their anodes was disorderly, the use of gel electrolyte hindered the formation of dendrites, hence improving its efficiency. It should be mentioned here that the improved efficiency was also due to their improvements of MnO_2_ cathodes by adding some additives to the MnO_2_ cathodes. Xiaotong et al. [18] synthesized a hollow MnO_2_ nanosphere cathode in an attempt to make a stable Zn-MnO_2_ battery. The battery also operated with better stability because the hollow MnO_2_ nanosphere structure favored the intercalation process of zinc ions. This research is considered very interesting in improving MnO_2_ cathode but to create a hollow nanoshere cathode requires a template which costs much more than the preparation of MnO_2_ cathode in general. In 2018 Mylad et al. [19] conducted a study on Zn-MnO_2_ by using ZnSO_4_/MnSO_4_ as electrolyte. it was found that ZnSO_4_ precipitated during discharge and it was reported that precipitation of the salt did not affect the chemical reaction in the battery. In the preparation of their anode, their use of Zn powder was more complicated than that of this study. It should be mentioned that efficiency of the battery of Mylad et al. was similar to that of the present investigation. It is also worth noting that the use of ZnSO_4_/MnSO_4_ as electrolyte can be an interesting choice since it is non-toxic and non-corrosive to electrical components.

**Table 2:** Some important test results of Zn-MnO_2_ batteries by current researchers.

Anode	Cathode	Electrolyte	Efficiency (%) *@100cycles	References
Zn electrodeposition on carbon cloth	MnO_2_	2 M ZnCl_2_ + 0.4 M MnSO_4_ (Gel electrolyte)	∼80%	[15]
	MnO_2_+PEDOT		∼99%	
Zn anode from Zn powder with binder	MnO_2_	2 M ZnSO_4_	∼99%	[16]
	MnO_2_	2 M ZnSO_4_ + 0.1 M MnSO_4_	∼97% more stable	
Zn electrodeposited on nanoplate anode	Carbon/CNT/MnO_2_ past	2 M ZnSO_4_ +0.2 M MnSO_4_ (hydrogel)	∼99%	[17]
Zn metal as anode	MnO_2_ nanosheets	1 M ZnSO_4_ + 0.2 M MnSO_4_	∼97%	[18]
	MnO_2_ nanorods		∼99%	
	MnO_2_ Hollow nanospheres		∼99% more stable	
Zn deposits on Cu plate Current density 0.1 A/cm^2^ with 0.5 M ZnSO_4_	MnO_2_	6M KOH + 40%ZnO	∼80%	This work
Zn deposits on Cu plate Current density 0.1 A/cm^2^ with 1 M ZnSO_4_			99.97% more stable	

In the development of Zn-MnO_2_ battery to have high efficiency, stability, long working life, and environmental friendliness, the battery should be made from inexpensive and readily available material which will result in a reasonable production cost, hence commercially inexpensive battery.

## 4 Conclusion

The concentration of the electrolyte and the current density utilized in zinc electroplating on copper substrate strongly influence the morphology of zinc deposits. Since the morphology of zinc had a direct effect on the performance of the zinc electrode, an increase in electroplating time in electrodeposition revealed that there was more sprouting of zinc which affects the battery's life. As a result, preparing the zinc electrode on a copper substrate using ZnSO_4_ concentration of 1 M and current density of 0.1 A/cm^2^ provided an orderly morphology with a higher current efficiency of 97.09%. This condition is optimal for zinc electrode on copper substrate in this study. The anode prepared under the optimal condition was tested and found to have 99.97% efficiency and operated with better stability. The battery efficiency depends not only on the morphology of Zn deposits but also on the type of electrolyte used. Therefore, an investigation into the improvement of electrolytes for the battery is strongly recommended.

## Declarations

### Author contribution statement

Nattaporn Chaba: Conceived and designed the experiments; Performed the experiments; Analyzed and interpreted the data; Wrote the paper.

Sutasinee Neramittagapong & Nawapak Eua-anant: Conceived and designed the experiments; Performed the experiments; Analyzed and interpreted the data; Contributed reagents, materials, analysis tools or data.

Arthit Neramittagapong: Analyzed and interpreted the data; Contributed reagents, materials, analysis tools or data.

### Funding statement

This work was supported by the Energy Conservation Foundation, Energy Planning and Policy Office, Ministry of Energy, Thailand. This work was also supported by the Graduate School of Khon Kaen University (Grant no. 58142102).

### Competing interest statement

The authors declare no conflict of interest.

### Additional information

No additional information is available for this paper.
